# Simultaneous detection of macroevolutionary patterns in phenotypic means and rate of change with and within phylogenetic trees including extinct species

**DOI:** 10.1371/journal.pone.0210101

**Published:** 2019-01-25

**Authors:** S. Castiglione, C. Serio, A. Mondanaro, M. Di Febbraro, A. Profico, G. Girardi, P. Raia

**Affiliations:** 1 Department of Earth Sciences, Environment and Resources, University of Naples Federico II, Napoli, Italy; 2 Department of Earth Sciences, University of Florence, Firenze, Italy; 3 Environmetrics Lab, Department of Biosciences and Territory DiBT, University of Molise, Pesche, Italy; 4 Dipartimento di Biologia Ambientale, Università di Roma Sapienza, Rome, Italia; CNRS - Universite de Pau et des Pays de l'Adour - E2S UPPA, FRANCE

## Abstract

Recognizing evolutionary trends in phenotypic means and rates requires the application of phylogenetic comparative methods (PCMs). Most PCMs are unsuited to make full use of fossil information, which is a drawback, given the inclusion of such data improves, and in some cases even corrects, the proper understanding of trait evolution. Here we present a new computer application, written in R, that allows the simultaneous computation of temporal trends in phenotypic mean and evolutionary rate along a phylogeny, and to contrast such patterns among different clades within the tree.

By using simulation experiments, we show the new implementation, names *search*.*trend* is as powerful as existing PCM tools in discerning macroevolutionary patterns in phenotypic means and rates, but differently from any other PCM allows comparing individual clades to each other, and provides rich information about trait evolution for all lineages in the tree.

## Introduction

Recognizing evolutionary trends in phenotypic means and rates has always been a major topic in evolutionary biology [1–4]. Phenotypic patterns such as Cope’s rule [5–7], the Island Rule [8–10] and morphological stasis [11,12] attract continuing interest from students of phenotypic evolution. Similarly, the rate of phenotypic change is intensely investigated [13–15]. The adaptive radiation theory predicts the rate should decrease over time [16,17]. However, it might be accelerated by the introduction of phenotypic innovations [18–20], by ecological release after mass extinctions [21,22], and by major tectonic and climatic events [23,24]. The detection of such temporal patterns in phenotypic means and rates is complicated by the presence of phylogenetic effects. Phylogenetically close species share similar phenotypes, meaning they resemble each other more than expected by chance. Hence, the magnitude and direction of trait change must be contrasted to null models which take phylogenetic relatedness into account. Phylogenetic comparative methods (PCMs) offer the opportunity to understand the tempo and mode of trait evolution while controlling for phylogenetic effects. Unfortunately, PCMs are not always well-suited to deal with extinct phylogenies, and most of those that are, do not allow comparing trends in rates and trait means between clades within a single tree. Still, most PCMs rely upon a priori, necessarily low-dimensional evolutionary models to be compared to each other. The recently developed *RRphylo* method Castiglione et al. [25] allows computing phenotypic evolutionary rates for all branches in the tree and phenotypic estimates at nodes (i.e. the ancestral states). It is especially meant to work with phylogenies of extinct species, and assumes no specific a priori evolutionary model, depending entirely on the distribution of phenotypes on the phylogeny. While this might complicate the straightforward interpretation of the tempo and mode of phenotypic change, *RRphylo* permits taking full advantage from fossil information, which is always welcome, since this provides better and more realistic interpretation of trait history than with phylogenies restricted to living species [26–29].

Here, we show a new implementation based on *RRphylo*, named *‘search*.*trend’* which allows computing simultaneously the temporal trends in phenotypic rates and means, and to compare trends among different clades within the phylogeny.

We tested *search*.*trend* performance by using simulations, measuring both Type I and Type II error rates. We provide the R code (*‘search*.*trend’*) and data for the method, and embedded it in the ‘gitHub’ version of the R package RRphylo (available at https://github.com/pasraia/RRphylo).

## Materials and methods

The *search*.*trend* function takes an object produced by the R package namesake function. Such *RRphylo* function computes rates and phenotypes for all branches and nodes of the tree, respectively, by applying normalized phylogenetic ridge regression [25].

The *search*.*trend* algorithm regresses the absolute value (i.e. the magnitude) of the phenotypic evolutionary rates calculated by *RRphylo* against their ages, meant as the distance of the branch from the tree root. A second, separate regression is performed between the vector of phenotypes (obtained by collating ancestral phenotypic estimates to trait values at the tree tips) and their ages. Throughout the rest of the manuscript, we refer to the former as the regression to test for the existence of a ‘trend’ in the rates, and to the latter as a test for the ‘drift’ in the phenotypic mean, over time. For both regression slopes (i.e. trend and drift), significance is assessed as the probability that the actual slopes differ from a family of 100 regression slopes (BM_slopes_) generated according to the Brownian motion model of evolution, by using the function *fastBM* in the R package phytools [30].

**Fig 1:**
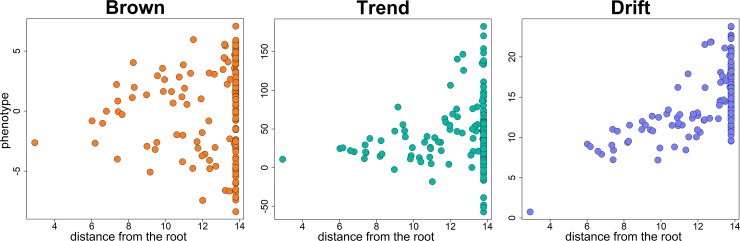
The distribution of phenotypes simulated according to the Brownian motion model of evolution (“Brown”). This same Brownian motion phenotypic vector is transformed by applying to it a trend for increasing phenotypic evolutionary rate towards the present (“Trend”) or increasing its phenotypic mean towards the present (“Drift”).

The Brownian motion has two free parameters, the phenotypic value at the tree root (herein named *rootV*) and the Brownian rate *σ*^2^. *RRphylo* estimates ancestral states (including *rootV*) as the products of the matrix of branch lengths multiplied to the vector of rates (the latter are normalized as to avoid extreme rate values, which makes ridge regression different from ordinary least squares regression [25]). By default, *rootV* is computed as the average value of the 10% most ancient tips in the tree, weighted by their squared distance from the root (meaning that older species have more influence on *rootV* estimation). This means that the estimation of unknown phenotypes is entirely dependent on the tree known (tip) phenotypes, rather than depending on the assumption of a particular mode of evolution, such as BM. Rather than a single rate such as in the Brownian motion, *RRphylo* assigns a rate to each branch of the tree, estimated via phylogenetic ridge regression [25,31]. Such ‘rates’ actually represent regression coefficients, describing the pace of phenotypic change between two consecutive nodes in the tree. As such, rates represent the phenotypic change per unit time between consecutive nodes in the tree. Hence, with a phenotype evolving according to the Brownian motion model, the magnitude of *RRphylo* rates increases with the distance from the root (i.e. towards the present) in keeping with the increase in phenotypic variance (Fig 1).

**Fig 2:**
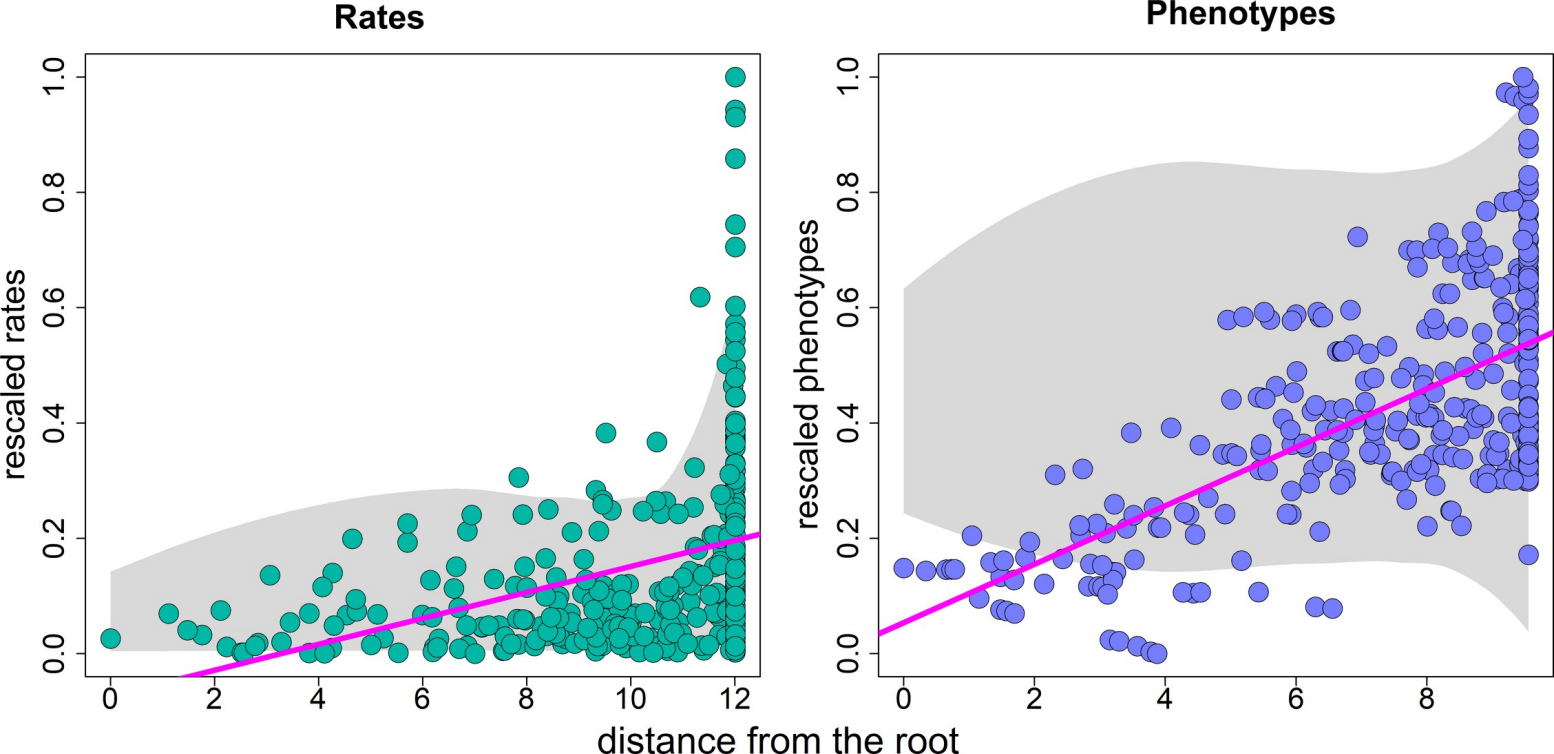
**Trend (left) and drift (right) regressions**. The green and blue dots represent the actual distribution of rates and phenotypes (respectively), obtained after applying a trend to the rate and a drift to the phenotypes, as illustrated in Fig 1. The gray shaded area represents the distribution of values (either rates, left, or phenotypes, right) generated under the Brownian motion model for the same tree. Notice that both rates and phenotypes are rescaled to the 0–1 range. Rates are logged before running the trend regression.

Ideally, when a positive trend in the rate of evolution towards the present applies (Fig 1, middle), the slope of the rates versus age regression would be larger than any BM_slopes_ (Fig 2). However, three sources of uncertainty impinge upon this ideal situation. First, since rates are proportional to the actual the phenotypic values rates must be rescaled into the 0–1 range before running regressions to make the real regression slope entirely comparable to BM_slopes_. Secondly, in the presence of a temporal trend in the evolutionary rates, *rootV* might assume an extreme value within the distribution of phenotypes, which will generate a heavily skewed distribution of *RRphylo* rates. Thirdly, the distribution of rates is influenced by variation in branching times across the phylogeny. Since closely related species tend to have similar phenotypes, evolutionary rates will be small where the tree is dense with species. This could produce a declining slope of the trend regression line if the dense part of the tree coincides with a single, large, recent clade, even under a regime of increasing rates over time. To account for these caveats, the rates versus age regression in *search*.*trend* runs with logged data (to reduce the skewness of the rate distribution). Still, to properly assess the direction of rate variation through time, *search*.*trend* checks whether the standard deviation of the rates in the branches falling in the first (older) half of the tree is significantly smaller than the corresponding figure for the second (more recent) half of the tree, as compared to BM simulations, which is expected to occur if a positive trend in the evolutionary rate is present in the data.

With the drift regression (phenotypes versus age, Figs 1 right and 2 right), the original phenotypes as well as those producing the BM_slopes_ should be rescaled to the 0–1 range as well, to account for the actual value range of the individual variables. For all the BM_slopes_, regardless of whether rates or phenotypes are used, simulated phenotypes are produced by imposing *σ*^2^ = 1 in *fastBM*.

### Simulation experiments

We performed a number of simulation experiments to assess the sensitivity of *search*.*trend* to both Type I and Type II error rates, and to the intensity of the simulated pattern. We started by producing 200 random, non-ultrametric trees by using the function *sim*.*bdtree* in the package geiger [32], setting the birth rate at 0.5 and the death rate at 0.2, but retaining only trees with at least 80 species. In each iteration, we started by simulating on the tree a phenotype evolving according to Brownian motion (BM), by using the function *fastBM* in the package phytools [30]. In producing the BM phenotypes the phylogenetic mean was randomly chosen from a uniform distribution spanning from -10 to 10 and the Brownian rate was randomly picked from a uniform distribution of 300 values spanning from 0.01 to 10. To assess the power of *search*.*trend* to recognize the actual phenotypic pattern, this Brownian phenotype was then transformed according to 1) a trend of exponential increase or decrease of the phenotypic variance over time (representing time-dependent changes in the phenotypic evolutionary rate, ‘trend’, Fig 1, middle) and 2) a phenotypic drift (Fig 1, right). In order to transform the BM phenotype, in the former case, the age distances from the tips to the root (the tip *times*) are elevated to the power *es*, and the resulting phenotype will thus be equal to *y* *(*times*^es)/*times*; where y is the original Brownian motion phenotype. With *es* = 1 the simulated phenotype represents the brownian motion, with *es* >1 the variance of the phenotypes grows exponentially with the distance from the root, and the converse with *es* < 1 (Fig 1 middle). To simulate a drift in the phenotypic mean over time, the vector of *times* was multiplied by a scalar *ds*, according to the equation y + times * ds, where *y* is the original BM phenotype. This way, with *ds* > 0 there will be a positive drift (an increase in the phenotypic mean over time) and the other way around with *ds* < 0.

**Fig 3:**
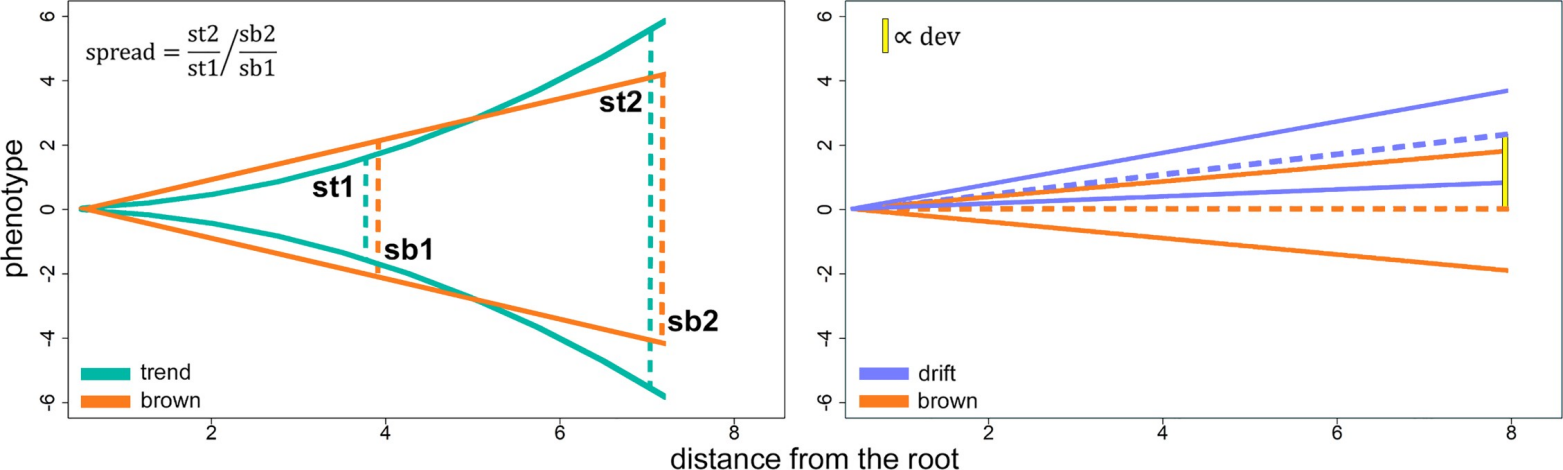
**The application of phenotypic drift (left) and trend in rate variation (right) develped by the RRphylo function setBM and used in the simulations described in this study**. For both transformations, a metric (dev for phenotypic drift, spread for rate trend) measures the deviation of the transformed phenotype from the Brownian motion model.

We assessed the sensitivity of *search*.*trend* to variations in either *es* and *ds* (setting the former to vary randomly between -1 and 3 and the latter to vary randomly in between -2 and 2). By using these parameters, we simulated both a trend-ed and a drift-ed phenotype on each of the 200 random trees. At each simulation, we recorded for both trend and drift regression the rank P of the real regression slope among the BM_slopes_, either the starting *es* or *ds*, the *σ*^*2*^ of the transformed phenotypes, and a metric representing the magnitude of the phenotypic deviation from Brownian motion as imposed by the *ds* (or *es*) transform. For the former, the metric (*dev*, Fig 3 left) represents the deviation of the phenotypic mean from the root value in terms of the number of standard deviations of the trait distribution (*dev* is zero under the Brownian motion). *dev* necessarily grows with the distance from the root and the (absolute) value of *ds*. In the case of ‘trend’, we computed the metric *spread*. It represents the ratio between the range of phenotypic values and the range of such values halfway along the tree height, divided to the same figure under Brownian motion (Fig 3 right).

After the simulations, we regressed the 200 P values against the corresponding *es* and *ds*, in order to find the specific values of the phenotypic transforms (named *es*_sig_ and *ds*_sig_, respectively) that mathematically coincide with significant Ps (that is P > 0.95 or P < 0.05, for either declining or increasing rates and phenotypic means). For the trend cases this generates an *es*_*sig*_*+* for P < 0.05 and an *es*_*sig*_*-* for P < 0.95. The same applies to *ds*_sig_ for the drift case.

We compared the performance of *search*.*trend* to existing methods to assess the presence of phenotypic drift and rate trends. The exponential change in phenotypic variance provided by *es* is similar to application of the delta transform [33]. We thus used the R package geiger function *fitContinuous* to fit and then compare to each other (by means of the likelihood ratio test) other the “delta” and “BM” model of evolution on Brownian motion phenotypes modified by applying *es*_sig+_ and *es*_sig-_. The same was repeated at *ds*_sig_ (again for both positive and negative drift patterns) to compare the BM and the “drift” model in *fitContinuous*’. Two hundred such comparisons were run. This allows comparing Type II error in both *fitContinuous* and *search*.*trend* at the two *es*_sig_ and the two *ds*_sig_ respectively. Eventually, we applied both *search*.*trend* and *fitContinuous* to 200 additional phenotypes simulated according to the Brownian motion (by using *fastBM*) in order estimate Type I error rates for both functions.

### Contrasting different clades to each other and to the rest of the tree

Differently from any existing PCM, *search*.*trend* is designed to contrast different clades within the tree to find significant differences in the pattern of phenotypic evolutionary rate and phenotypic mean change over time. In the case of phenotypic drift, individual clades are tested for the hypothesis the drift slopes do not depart from the Brownian motion expectation. However, in the case of trend regressions the actual regression slope depends on the relative position (age) of the focal nodes respective to the root, given the exponential nature of phenotypic variance change in time. Because of this, for the trend case *search*.*trend* compares estimated marginal means predictions from the linear regressions (of the rate versus age regression) by using the function *emmeans* embedded in the package *emmeans* [34].

To test the power of the group (clade) comparison module of *search*.*trend*, we used the same trees as in the simulation experiments described above. We first simulated a BM phenotype. Then, we randomly selected a node in the tree subtending to twenty species at least and applied to this clade twice as much as *es*_*sig*_ or *ds*_*sig*_ (separately) depending on whether trend or drift are being tested. For the latter, since the actual drift depends on both *ds* and the total time of evolution (i.e. the height of the tree) we further accounted for the differences between the tree height and the subtree (the drift-ed clade) height by multiplying *ds* for the ratio between the two heights. This means individual clades being tested for trend are transformed according to the equation *y* *(*times*^2es_sig_/*times*); those being tested for drift are transformed according to the equation y + times * 2ds_*sig*_ * H_*ratio*_, where *y* is the BM phenotype, the vector *times* is the vector of tip to root distances, and *H*_*ratio*_ is the ratio of tree height to the subclade height.

**Fig 4:**
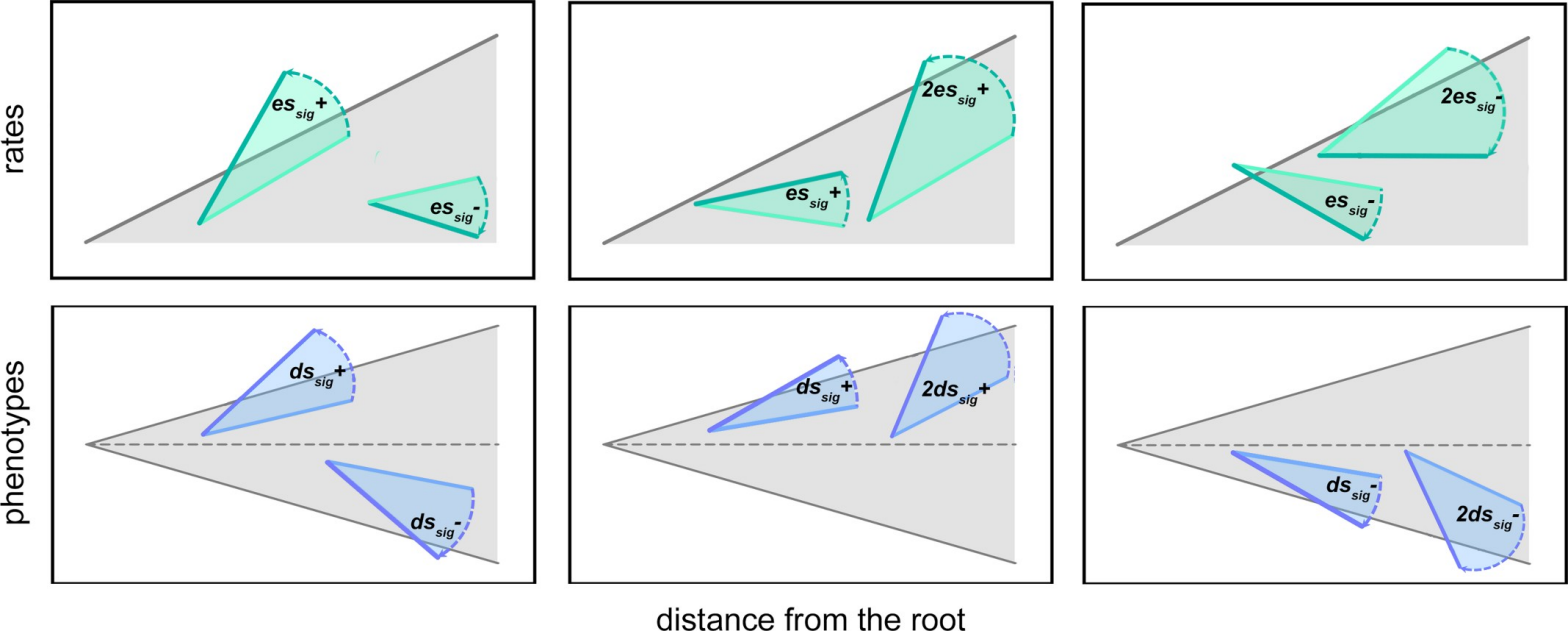
Illustration of the simulation experiments performed to test the power of *search*.*trend* function to correctly identify clades evolving at different regimes from the rest of the tree. The gray shaded area represent the idealized distribution of phenotypes and rates according to the Brownian motion model of evolution. In the simulations, individual clades are selected and then transformed by imposing a distinctive phenotypic rate regime (upper row) or phenotypic mean change regime (lower row).

To test the power of *seach*.*trend* to find significant differences in the patterns of rate or phenotypic mean change over time we separately selected two non-overlapping nodes in the tree subtending to at least 20 species each, and proceeded as above, applying twice as much as *es*_*sig*_ or *ds*_*sig*_ for the trend and drift case, respectively. In both the single-clade and two clades modified experiments, the sign of the *es*(*ds*) transform was random, meaning the that focal clades phenotypes might be altered either in the same, as well as in opposing directions (Fig 4).

Eventually, we tested the unmodified, Brownian motion phenotypes for the same set of subclades, to find the frequency of false positives (i.e. clades that appear as evolving differently from the rest of the tree or from each other) restituted by *search*.*trend*.

## Results

### Simulations

**Fig 5:**
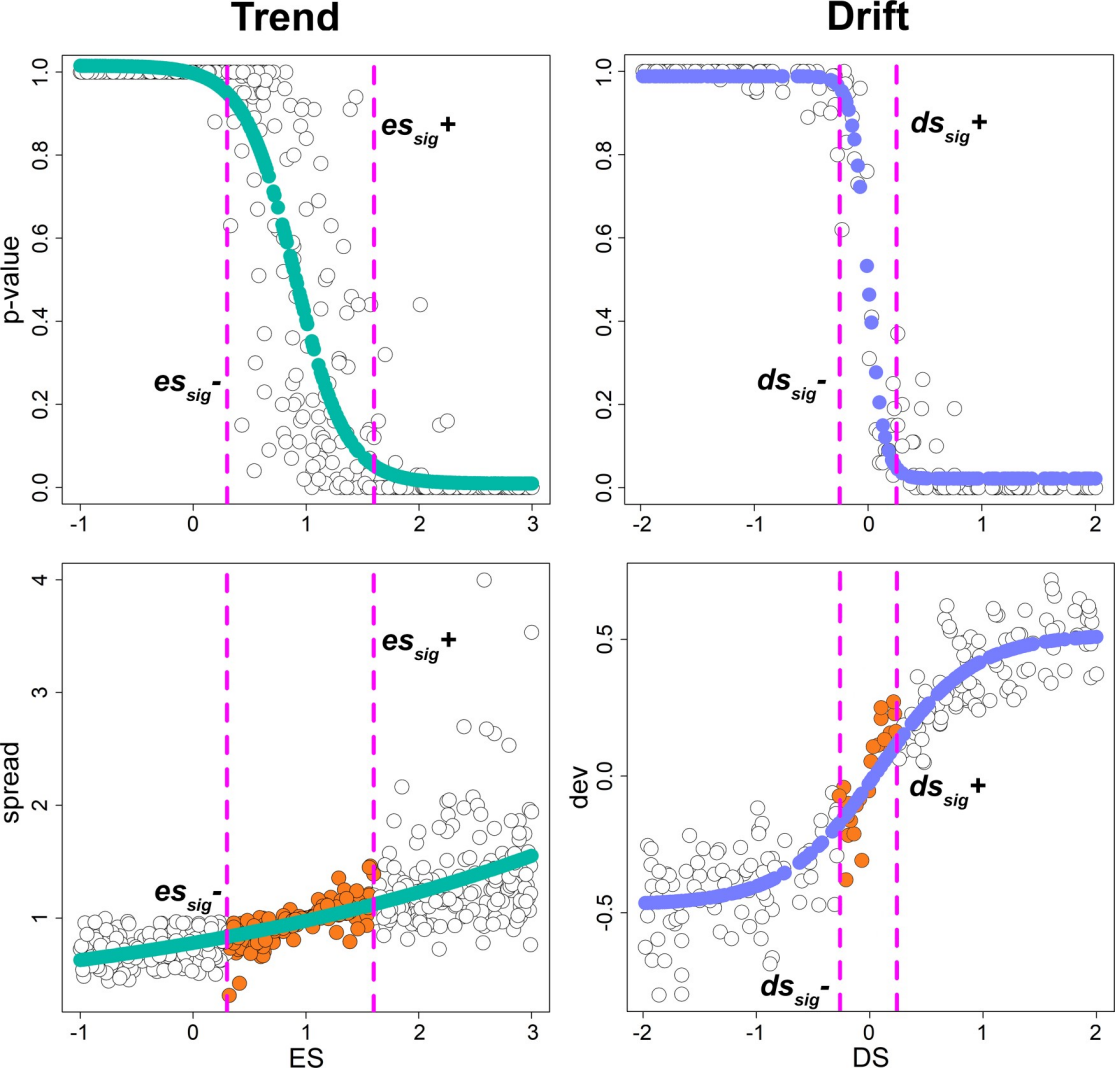
**Upper row, the relationship between the intensity of the simulated phenotypic pattern (obtained applying the *es* and *ds* transforms) and the power of *search*.*trend***. Lower row, the effect of the *ds* and *es* transforms on the original (untransformed) Brownian motion phenotype. For the phenotypic drift (lower row, right) we plotted the standard phenotypic deviation (*dev*) from the root at *ds*_*sig*_ (i.e. at *ds* = 0.25 and *ds* = -0.25). For the rate trend (lower row, left), we plotted the *spread* metrics after applying the *es* transform at *es*_*sig*_ (orange full dots, *es* = 0.3 and 1.6). The orange dots represent the values of *es* or *ds* either where *search*.*trend* finds no significant differences from Brownian motion.

**Fig 6:**
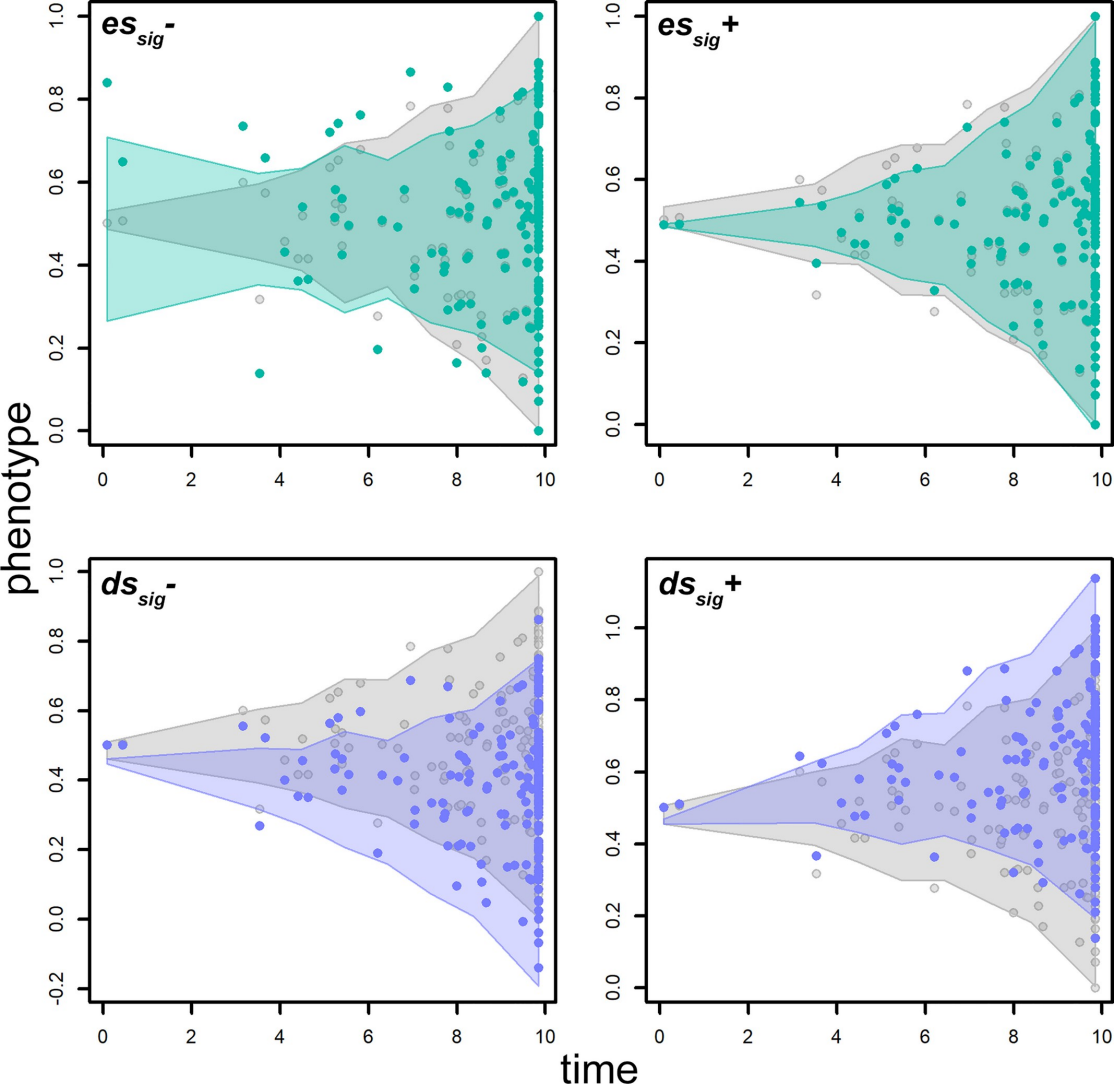
The change in phenotypic values occurring by transforming a Brownian motion generated phenotype (gray dots) by applying a trend in the rate of evolution (upper row, green dots) or a drift to the phenotypic mean (lower row, purple dots). The shaded areas represent the distribution of values generated after 100 random iterations. The gray shaded area represents the distribution of Brownian motion simulations. The green shaded area represents the distribution of trend-ed phenotypes. At *es*_*sig*_*-* the phenotype is simulated according to a declining rate of phenotypic evolution. On the opposite, the rate increases at *es*_*sig*_+ (right upper corner). At *ds*_*sig*_*-* the phenotypic mean becomes smaller over time (left lower corner), the opposite applies at *ds*_*sig*_+ (right lower corner).

The *search*.*trend* function performs well in terms of finding the simulated patterns. As applied to phenotypes simulated according to the Brownian motion the function error rate is close to the nominal level (5%) in both the trend and the drift cases (Table 1). The distribution of significance values plotted against the intensity of the applied transform (i.e. either *es* or *ds*, Fig 5, upper row) indicates *search*.*trend* gives significant results at *ds*>.25 and *ds*< -0.25 and *es* >1.6 and *es* < 0.3 for the drift and trend case, respectively. These values thus correspond to the *ds*_*sig*_ and *es*_*sig*_ pairs, respectively. A *ds*_*sig*_ of 0.25 (or -0.25) the *dev* statistics is ± 0.18, which means the phenotypic mean deviates some 0.18 standard deviations from the root value per unit time, which is a modest phenotypic drift (Fig 6). At applying a regime of rate variation over time (the trend case) the spread statistic (the ratio between the range of phenotypic values and the range of such values halfway along the tree height) at *es*_*sig*_ 84% to 112% of the spread which occurs under the Brownian motion model (Fig 6). As a guidance, we empirically found this is equivalent to apply modest delta transform to the tree with delta values of 0.3 and 1.4, respectively (S1 Fig).

**Table 1:** Performance of *search*.*trend* for both rate trend and phenotypic drift recognition, compared to geiger’s *fitContinuous* function.

	fitContinuous			search.trend		
Phenotype	BM	Delta	Drift	BM	Trend	Drift
Brownian Motion	0.95	0.05	-	0.93	0.07	-
Trend+	0.26	0.74	-	0.21	0.79	-
Trend-	0.32	0.68	-	0.02	0.98	-
Brownian Motion	0.97	-	0.30	0.98	-	0.02
Drift+	0.30	-	0.70	0.26	-	0.74
Drift-	0.22	-	0.78	0.28	-	0.72

The simulated phenotypes correspond to Brownian motion, a positive trend in the rates at es_sig+_ (Trend+), a negative trend in the rates at es_sig-_ (Trend-), a positive drift in the phenotypic mean at ds_sig+_

(Drift +), a negative drift in the phenotypic mean at ds_sig-_ (Drift -). The numbers refer to the percentage of correct pattern identification. For instance, with a phenotype evolving according to the Brownian motion model the *fitContinuous* power is 95% and the error rate (the percentage of iterations where the delta model was recognized better than BM) is 5%. The corresponding figures for *search*.*trend* are 93% and 7% (first row).

Despite the simulated trees differ considerably from each other in terms of size (the average number of tips is 165, range 128–226) tree size bears no influence on the power of *search*.*trend*. In the case of trend-ed phenotypes, the relationship between P (the rank P of the real regression slope among the BM_slopes_) is not significant (p = 0.794, R^2^ = 2.5 * 10^−4^). The same applies to drift-ed phenotypes (p = 0.160, R^2^ = 0.03).

As compared to geiger’s package *fitContinuous*, *search*.*trend* is at least as powerful and accurate. For the trend-ed phenotypes, simulated at *es*_*sig*_, *search*.*trend* works better than *fitContinuous*, especially when negative trends in the rate over time are simulated (Table 1). The power of the two functions is very similar when drift-ed phenotypes (as *ds*_*sig*_) are simulated.

### Contrasting different nodes

*search*.*trend* applied to nodes evolving at different regimes from the rest (Brownian motion) of the tree is able to recognize the simulated patterns. When a single node is transformed at twice *ds*_*sig*_ over *search*.*trend* successfully recognized the phenotypic transformation in > 80% of the cases (Table 2). With the trend case, this percentage rises above 90%. The corresponding Type I error rates (instances of reportedly significant phenotypic change on nodes which were left, in fact, untransformed) is as low as 6 (trend) and 2% (drift). When two different, non-overlapping clades are transformed at one time, the function power still remains close to 90% if the two nodes are transformed in opposite directions (i.e. by applying *es* or *ds* transformations with different signs, Table 2) but becomes much less powerful if the two clades are transformed in the same direction (Table 2). Under all conditions, the Type I error rate remains close to the nominal alpha level (Table 2). This is particularly robust considering individual node heights vary in between 20 to 97% of the tree height and might include as many as 20 to 84 species.

**Table 2:** *search*.*trend* performance as assessed by transforming individual clades (either one clade or two clades) within the tree.

	One clade	Two clades		
		Tested Individually	Against each other	
Simulated phenotype			Opposite sign	Same sign
**BM**	0.060	0.070	0.060	
**Trend+**	1.000	0.984	1.000	0.275
**Trend-**	0.920	0.875		
**BM**	0.020	0.010	0.050	
**Drift+**	0.882	0.935	0.956	0.148
**Drift-**	0.857	0.882		

The distribution of Type I and Type II error rates for *search*.*trend* assessed on complex phenotypes simulated to exhibit different patterns in different parts of the tree. Two kinds of patterns are simulated, indicating either an increase (indicated with plus symbol), or a decrease (indicated with the minus symbol) in either ‘trend’ or ‘drift’, pertaining to either a single, or two clades within the tree.

## Discussion

Fossil information provides fundamental insight about the evolution of traits, so much that the absence of such information might lead to erroneous inference and low power in detecting the real signal in phenotypic evolution [26,35,36]. Secular patterns in specific traits such as body size and the degree of encephalization are given exceptional attention by students of trait evolution. Yet, unfortunately, they are often investigated without using PCMs [6,37–40]. Even when fossil phenotypes and phylogeny are considered, comparisons between clades are usually not performed, and the simultaneous estimation of evolutionary trends in phenotypic means and rates are, to our knowledge, even rarer. The method we propose here, *search*.*trend*, provides a way to estimate straight away the existence of such patterns, and to compare individual clades within the to each other. The *search*.*trend* function demonstrated to have high power and consistently low Type I error (Table 1), and to be at least as accurate as geiger’s *fitContinuous* suite of functions. The latter is admittedly much faster than *search*.*trend* (we averaged the time to completion on the same phenotype and tree for the two functions over ten iterations getting an mean of 2.55 seconds for *fitContinuous* and 20.19 seconds for *search*.*trend*). Yet, *search*.*trend* provides information about the direction and intensity of the two patterns, writes figures as pdf files to let the experimenter gauge the exact meaning and distribution of the patterns which are found, provides confidence intervals around the estimates for both rates and phenotypes, restitutes the phenotypes and rates per age and per branch (which allows further inspection of the distribution of such metrics per clade and per age and, if desired, the application of regression models different from the linear model which the function uses by default) and, on top of all, *search*.*trend* allows comparing directly individual clades within the phylogeny with each other and against the rest of the tree. This latter, fundamental feature means that individual clades can be compared to each other for the existence of either drifts or trends in the phenotypic mean and variance, respectively, even when the actual phenotype is a complex admixture of different evolutionary regimes (Table 2). We found that *search*.*trend* has good power in finding the designed pattern and shows small Type I error rates even under small deviation from Brownian motion (Table 1). The group (clade) comparison module of the function provides evidence that it effectively recognizes whether two clades in the tree evolve into different directions (either in terms of phenotypic mean or change in the evolutionary rate) when they are designed to be (Table 2, ‘opposed sign’). When the two selected clades do evolve into the same direction (Table 2, ‘same sign’), the function power to detect deviations of these clades from the Brownian motion decreases dramatically. However, rather than a limitation, this depends on the fact that when two clades in a tree are simulated to evolve according to a certain pattern in the phenotype (or in the rate either) the original BM phenotypic pattern of the tree as a whole is erased altogether (Table 2, ‘same sign’). We deliberately chose to build greatly variable random phenotypes and trees. In our simulations, tree size is 165 species on average (range 129–203), the selected nodes within these trees subtend to clades which include 38 species on average (range 20–84), and they are some one quarter the height of the tree they belong to (average = 28%, range 9%-97%). The starting value of the BM phenotypes (which is allowed to vary in between -10 and 10) has no influence on the function performance. Similarly, the starting Brownian rate does not influence performance. It is noteworthy that after transforming the BM phenotype by applying the *es* (or *ds*) transform, the actual Brownian rate covers 10 orders of magnitude (S2 Fig), still indicating the initial Brownian rate has no bearing on *search*.*trend* functioning.

Fossil information provides unique opportunity to look at phenotypic variation in the past and its change over time. While the relevance of such information to the proper understanding of trait evolution is well known, its full integration to the vast and powerful array of PCMs is limited by the unease of most PCMs to deal with paleontological phylogenies, and their limited description of macroevolutionary patterns. Here we provide a new, powerful addition to the existing PCM toolbox, which is appropriate to use when a full description of macroevolutionary patterns as captured by paleontological data and tree, and the simultaneous comparison between clades within the tree is the goal.

## Supporting information

S1 Fig Delta transformations to be applied in order to derive a phenotypic vector having as much spread as es_sig_- (left) and es_sig_+ (right).
(TIF)

S2 Fig P-values plotted against the Brownian rate (sigma^2^) for both ‘trend’ and ‘drift’ cases. Vertical dashed lines mark significant p-values. Orange dots represent the non-significant simulations (i.e. phenotypes recognized to evolve according to the Brownian motion).
(TIF)

S1 File R code. The computer code, written in R, to perform the set of simulations illustrated in the manuscript.
(R) Click here for additional data file.
