# Supplementary figures and images for: Simultaneous detection of macroevolutionary patterns in phenotypic means and rate of change with and within phylogenetic trees including extinct species
Source: PLoS One. 2019 Jan 25;14(1):e0210101. doi: 10.1371/journal.pone.0210101 (PMC6347132; doi:10.1371/journal.pone.0210101)

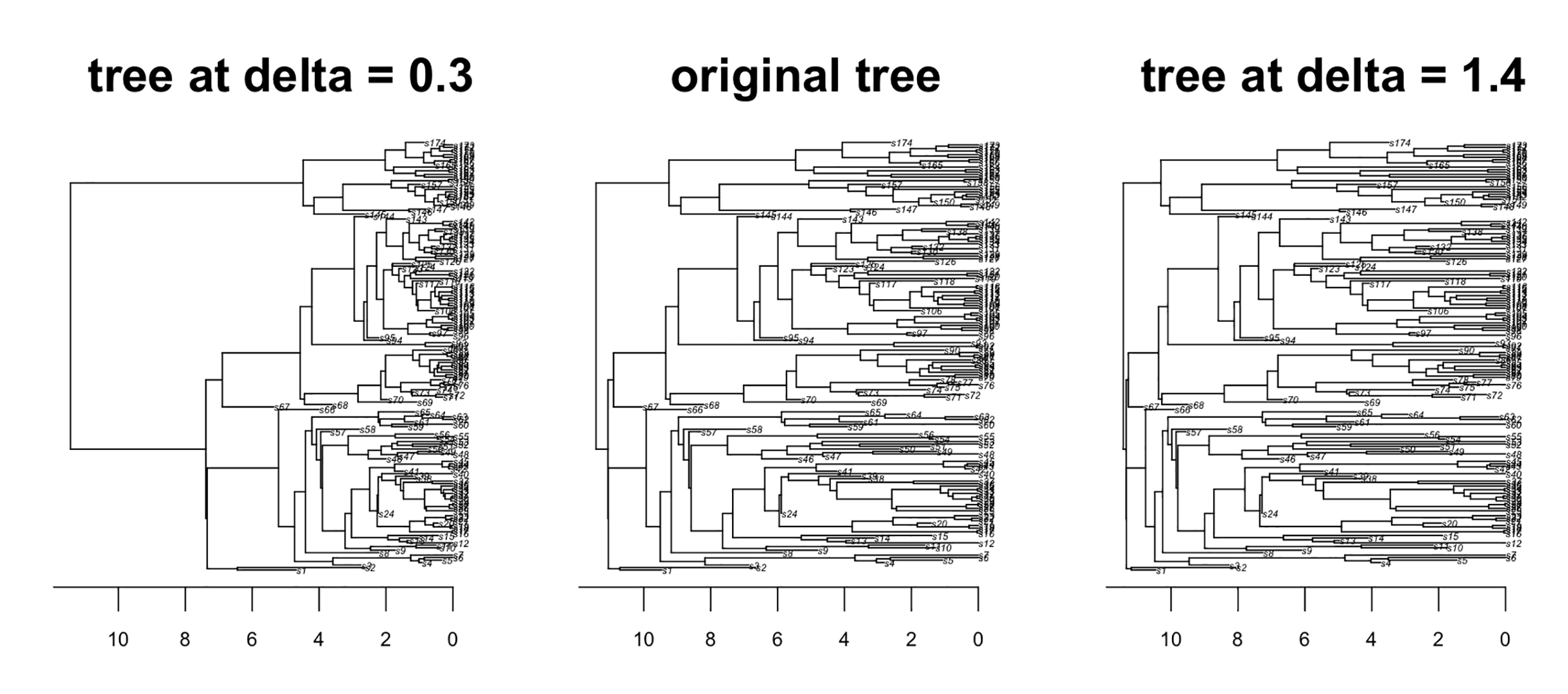

Supplement: S1 Fig — Delta transformations to be applied in order to derive a phenotypic vector having as much spread as essig- (left) and essig+ (right). (TIF) [file pone.0210101.s001.tif]

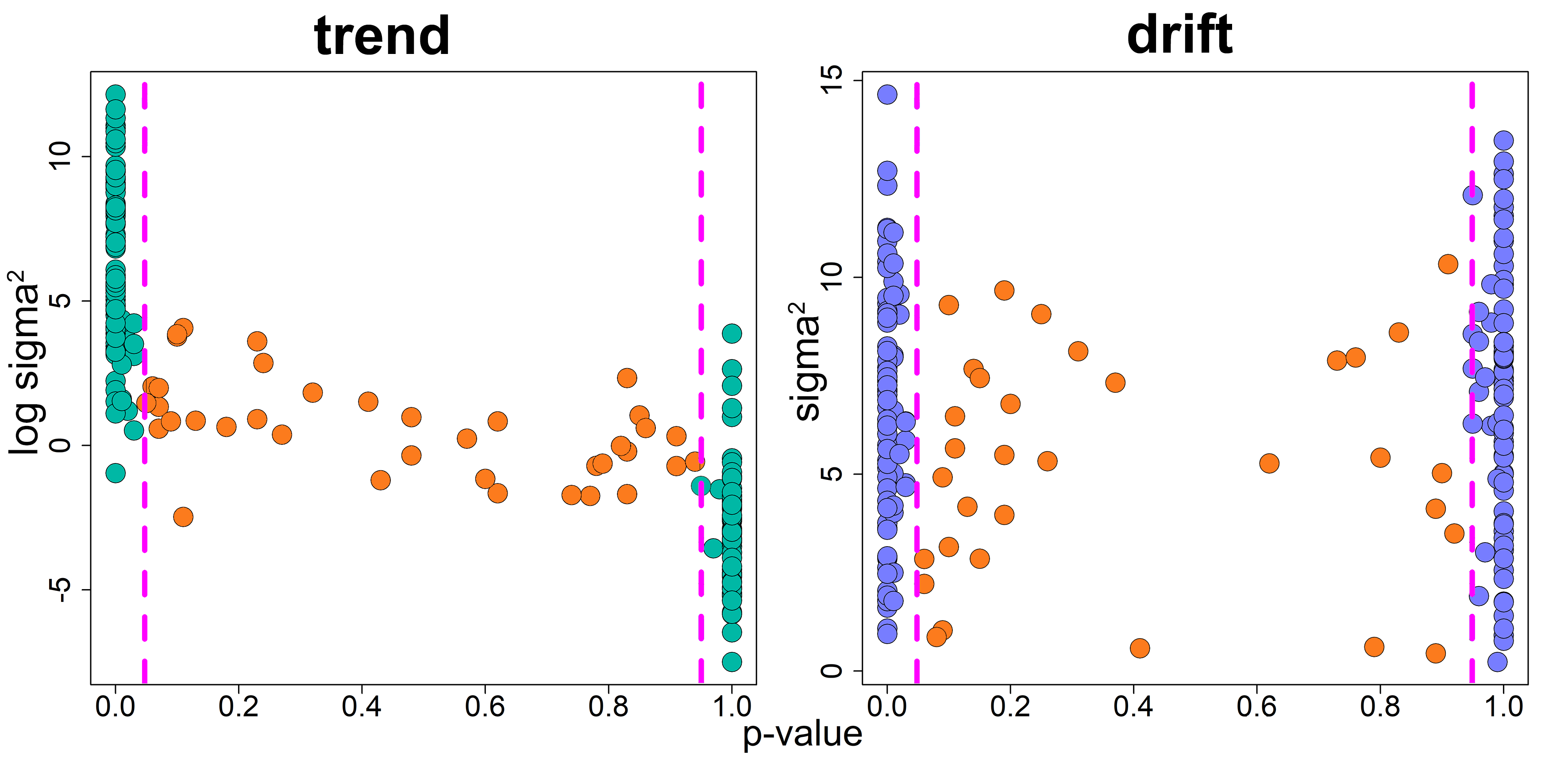

Supplement: S2 Fig — Vertical dashed lines mark significant p-values. Orange dots represent the non-significant simulations (i.e. phenotypes recognized to evolve according to the Brownian motion). (TIF) [file pone.0210101.s002.tif]
